# Sequelæ of Operations on the Bile Passages

**Published:** 1907-06-15

**Authors:** G. H. Makins

**Affiliations:** Surgeon to the Hospital


### Hospital Clinics.

### SEQUELÆ OF OPERATIONS ON THE BILE PASSAGES.

By G. H. MAKINS, C.B., F.R.C.S., Surgeon to the Hospital.

A Clinical Lecture on some Cases in Illustration, delivered at St. Thomas's Hospital.

Gentlemen,—In the course of the routine work of the hospital you see many operations performed for affections of the biliary passages. Such operations are undoubtedly among the most successful in the whole range of surgery, and, considering their magnitude, are unusually free from risk.

This freedom from immediate danger depends on the fact that the necessarily extensive manipulations are carried out in a region of special safety, in that they are made practically without the area occupied by the intestines. When the field of operation is properly packed off, the intestine, with the exception of the immobile second portion of the duodenum, is entirely excluded from view and from the possibility of infection; there is consequently no tendency to the diffusion of infective matter, which may be set free during the operation, into the area occupied by the mobile viscera. Of late years, moreover, the free access which is gained to the parts concerned, as provided by the incision designed by Mr. Mayo Robson, allows the more dangerous part of the operations to be effected practically without the confines of the abdominal cavity.

It is, however, perhaps more readily assumed than is justifiable that patients undergoing these operations are immediately and permanently relieved of all their troubles.

Experience shows that this is hardly a fact, and considering that the operations are performed for diseases which have often reached a late stage of their development and seriously involved surrounding structures, an absolute cure is hardly to be expected. The immediate relief afforded is, however, so strikingly satisfactory, both to the patient and to the surgeon, that the occasional after-conditions have perhaps not been so fully dwelt upon as could be desired. From the point of view alone of what we can promise prognostically, it is important to look the subsequent possibilities fairly in the face.

I propose, therefore, to devote this lecture to the consideration of three cases in which secondary interference has been required to relieve conditions following primarily successful operations.

The first two cases are illustrative of two forms of biliary fistula, the persistent and the recurring.

Case 1.—M. A. S. Æt. 58. Laundress.

The patient was admitted to Elizabeth Ward in July 1904, suffering from obstructive jaundice. The jaundice was extreme and the stools were "white." There was evidence of a very feeble heart, but none of valvular disease.

On July 25 a cholecystostomy was performed; a distended gall-bladder was found, and fifteen stones were removed from the bladder and common duct. As far as I could ascertain, the ducts were then free.

The patient was very ill for some days after the operation, suffering from ether-bronchitis and the results of her weak heart; but with care she steadily improved, the jaundice disappeared, the motions became properly coloured, and at the end of a month she was discharged to go to a convalescent home.

Seventeen days later a recurrence of the jaundice took place, and the healed wound burst open, a quantity of bile being discharged spontaneously.

The patient was then readmitted, suffering from jaundice, clay-coloured stools, some pain, occasional rigors, and attacks of vomiting. She remained in the hospital four months, during which time there were variations in degree of the symptoms, and on one occasion the sinus was enlarged. No further extensive operation was undertaken, in view of the fact that the patient had nearly died at the time of the first operation, and that her general condition was now less satisfactory than before. For the next twenty months she was in different infirmaries with a persistent biliary fistula, and once for a short time in the London Hospital, but no further treatment was deemed advisable.

In October of this year, feeling that she had now survived two years in a wretched condition, while she had not materially lost in general strength, I sent for her again, with the determination again to explore the bile-passages and see if anything further could be done.

On admission the general nourishment was fair, the biliary fistula poured forth a large quantity of foul-smelling bile, and there was great irritation and dermatitis of the surrounding integument. No bile in the urine, no jaundice, but stools slate-coloured. An attempt was made to improve the condition of the surrounding skin by painting with hard paraffin ointment and the application of a "Colt's tube."

Pulse feeble, at right wrist scarcely perceptible; no valvular murmur; heart sounds weak, but cardiac action regular. No albuminuria.

On December 4 the former Robson's incision was reopened. The liver was found very firmly adherent by its anterior edge to the abdominal parietes, while adhesions of the omentum, colon, and duodenum, completely buried and obscured the bile passages.

The adhesions were separated, and the ducts and gall-bladder exposed. The foramen of Winslow was completely obliterated. On investigation three gall-stones were discovered—one in the common hepatic duct, one completely obstructing, and fixed just below the commencement of, the common duct, and one at the termination of the common duct, but palpable from the outer margin of the duodenum.

The common duct was now incised over the upper of the two stones, the stone removed, and the duct found to be wide enough to admit the little finger. The stone in the hepatic duct was readily removed, but the lower stone was so firmly fixed that it had to be crushed with the forceps and removed in pieces with the forceps and spoon.

A drainage-tube was inserted and sewn into the partly sutured opening in the common duct, and the gall-bladder was then rapidly removed.

The abdomen was closed in the usual manner.

The after-course has been very satisfactory. The patient did not suffer so much as from the primary operation, the previous stinking bile became sweet, and after fourteen days of free discharge has become very slight in amount. No further trouble has been experienced with the skin, the stools have become normal in colour, and, except for occasional sickness and pain in the abdomen, there have been no troublesome symptoms.

At the present time the wound is practically closed, and the patient is leaving the hospital at the end of thirty-two days for a convalescent home.

Case 2.—S. W. Æt. 43. Married.

Admitted in July 1904, suffering from pain in the right side of the back and occasional attacks of diarrhœa for five years. In December 1903 she had a severe attack of pain in the epigastrium, right hypochondriac, and iliac regions, but not accompanied by vomiting or rigor. There was no jaundice, and, as before, diarrhœa accompanied the attack of pain.

Pain and tenderness were present in July over the situation of the gall-bladder, but no tumour was discoverable.

On June 11, 1904, a cholecystostomy was performed. The gall-bladder was found much enlarged and thickened, full of mucus, and two gall-stones the size of gooseberries were removed. The common bile-duct was free. After the removal of the stones a small quantity of bile escaped. About half the enlarged gall-bladder was cut away and the remainder stitched to the skin.

Bile was discharged in some quantity for some weeks, but the fistula closed, and the patient left the hospital. In September she returned with a discharging fistula, from which some silk stitches were removed, and she then left the hospital apparently well. A slight discharge of bile continued, however, until March 1905, when the fistula closed.

During the past eighteen months, however, she has again been suffering with recurring attacks of pain in the region of the gall-bladder, becoming more frequent, even once a fortnight, and on several occasions bile-stained mucus has been discharged from a small opening formed spontaneously in the centre of the old cicatrix. No vomiting, diarrhœa, or jaundice has accompanied the process.

On admission the wound was closed, and there was nothing palpable beyond a gap in the parietes in the situation of the gall-bladder. Urine normal. General condition good.

On December 3 the old incision was reopened; dense adhesions connected the anterior margin of the liver with the abdominal parietes, and colon and omentum were adherent at the under-surface and neck of the gall-bladder, which latter had again attained a large size, in spite of half having been removed at the previous operation.

No obstruction or stone was discovered in the common duct, the cystic duct was very much thickened, and the lumen was diminished to the size of a fine probe and tortuous. The gall-bladder was removed.

The patient has made a good recovery, interrupted only by a stitch abscess, which opened spontaneously on the eighth day. There was some pain preceding this occurrence, but no further evidence of trouble, and on the twenty-seventh day the patient left for her home.

These two cases well illustrate the two forms of fistula I have mentioned, and before noting any special features in them it will be well to recall the recognised causes of the condition. These causes may be found in the gall-bladder, the cystic duct, or the common bile duct.

1. *Condition of the Gall-Bladder.*—(*a*) Fixation of its fundus to the abdominal parietes, especially in a low position; (*b*) fixation of the gall-bladder by adhesions to neighbouring viscera; (*c*) an abnormally enlarged gall-bladder, in the process of cicatrisation and contraction, becomes sometimes left with so great a redundancy of lining mucous membrane that the latter may prolapse from the cholecystostomy opening and prevent the possibility of spontaneous adhesion of the lips.

2. *Conditions of the Cystic Duct.*—(*a*) Permanent inflammatory thickening of the lining mucous membrane; as a temporary condition this is often met with as a result of the manipulations necessary for the removal of calculi, and is evidenced by a temporary cessation of the flow of bile; (*b*) a gall-stone may have been left; (*c*) the duct may be strictured or actually obliterated, as a result of the cicatrisation of the ulcerated surface produced by the impaction of calculi.

In any of these cases the discharge will naturally be simple mucus and not bile, but it may be persistent, or spontaneous discharges may recur from time to time.

3. *Conditions of the Common Duct.*—Persistent or recurring obstruction due to (*a*) a retained calculus; (*b*) stricture developing at the seat of impaction of a calculus, or at the papilla, in the second case possibly as the result of duodenal disease; (*c*) growths within the duct, especially at its orifice; (*d*) pressure on the duct from without, as in calculus, inflammatory conditions, or new growth of the pancreas.

It is well to bear in mind that the existence of a calculus in the bile duct in any of the above conditions may be merely casual, and in no sense causative of the biliary obstruction.

To return to our two cases. In case 1, I think there can be no doubt that gall-stones were left at the first operation, although not at that time of a size to give rise to complete obstruction. The surgeon may reasonably be blamed for such an occurrence, but it must be allowed that stones are not always easy of detection, and the accident may happen in several ways. Thus, stones may be secreted in pouches or folds of the mucous membrane forming as the duct contracts after distension has been relieved by incision; stones in the hepatic duct may escape detection at the time of operation, and later descend into the common duct and cause obstruction. Beyond this, undetected stones at the time of operation may be small and later grow to a sufficient size to cause complete obstruction, a process often favoured by the septic condition of the ducts which may exist. I believe the latter explanation to have been the correct one in the case under consideration.

Other difficulties exist; thus stones in the hepatic or common bile duct are often absolutely latent as far as producing clinical signs of their existence, while, on the other hand, attacks of colic of the classical type by no means always prove the existence of calculus; thus such attacks are observed where obstruction is due to kinking of the ducts and consequent obstruction; while even when stones do exist and have caused colic, hæmorrhage may occur followed by coagulation of the blood, and a recurring attack of colic may be due to the passage of a clot and not a calculus. Firm defibrinated clots are occasionally discovered in the gall-bladder in cases of cholecystostomy which could well give rise to such attacks. Again, difficulty of an opposite character may sometimes confront the surgeon even in the course of an operation, in that a gall-stone may be closely simulated by a hard lymphatic gland in the gastro-hepatic omentum during the exploration of the upper part of the common bile duct, or by hardened areas in the pancreas when the lower portion is under examination.

The occurrence of these difficulties should only teach the lesson that in all cases, even when we apparently have a simple uncomplicated gall-bladder full of stones, the common bile duct and its extensions should be thoroughly explored—a procedure of comparative ease to-day, when the incision already mentioned, combined with rotation of the liver, is employed.

The second case calls for little elaboration in addition to the history I have already read and the remarks I have made as to the causes of biliary fistula, which show two of the causes (external adhesions and narrowing of the cystic duct) to have been present and probably to have worked in concert. The case offers a typical instance of the recurrent fistula.

I will now pass to the third case, which illustrates another possible sequela of disease of the biliary passages and operations for its relief.

Case 3. M.A.S. Æt. 51. Female. Married.

The patient had enjoyed good general health, except for the occurrence of attacks of biliary colic accompanied by jaundice. These had been occurring with increasing frequency up to July 1904, when she came into the hospital and I performed a cholecystostomy. At that operation a small contracted gall-bladder was found, and a single stone about the size of a cherry, which had probably caused obstruction of the "ball-valve" character. The common bile duct was free.

On the nineteenth day the fistula had closed and the patient left the hospital well. Since then occasional dull aching pain in the epigastrium, right hypochondrium, and radiating towards the left shoulder, has been persistent; of late this has been worse, the pain extending to the back of the neck and even to the top of the head. The pain does not seem to have had any very definite relation to the ingestion of food, and there has been no vomiting; constipation has been troublesome.

Two years and four months after the operation she was readmitted to hospital. She was thin, although not emaciated, complained of pain as above, also of heaviness and distension after taking food, and of constipation. There was no vomiting.

Slight tenderness could be elicited on pressure over the lower part of the old cicatrix. The stomach appeared to be dilated, and on the administration of a seidlitz powder showed up well below the umbilicus. It was assumed that pyloric obstruction due to adhesions existed, and on opening the abdomen these were found around the pylorus, and, to a considerable extent, obliterating the lesser sac of the peritoneum. The stomach was much dilated, and, therefore, a posterior gastro-enterostomy was performed.

The result of the anastomosis was on the whole satisfactory, and the patient left the hospital very much improved, although still complaining occasionally of pain between the scapulæ and in the left shoulder.

The development of adhesions is not to be too readily ascribed to the performance of operations in this region, since these commonly exist already when the necessity for interference arises; the separation of adhesions during the performance of operations does, however, tend to spread the area already occupied by them, whatever care be exercised by the surgeon, and hence they form a considerable element in the definite prognosis. There is no doubt that in one form or other they form the most fruitful cause of after-troubles.

Allusion has already been made to the influence of adhesions in distorting the bile passages and preventing the re-establishment of the normal flow within them, and in the case last related we have an example of their evil effect on surrounding viscera. In this instance the stomach was the organ mainly concerned, but the colon, duodenum, or other parts of the small intestine may suffer in like manner, a certain degree of obstruction being developed capable of causing pain, flatulence, and constipation.

The last question to be dealt with is as to how these sequences of operations are to be, as far as possible, minimised. The importance of not overlooking stones, and the various difficulties which may accompany the search for them, have been already sufficiently dwelt upon. It remains shortly to consider how recurrent troubles connected with the condition of the gall-bladder and the presence of adhesions are to be best avoided.

With regard to the former, the simple expedient of removal of the organ is the best practice, and one steadily gaining in favour. Clinical observation has long shown the gall-bladder not to be an essential part of the biliary apparatus, since a closed sac containing mucus, which has been known to exist for some time without any appreciable effect *per se* on the patient, has been commonly found at operations and in the post-mortem room. For some years, also, the result of operations has shown that the removal of the gall-bladder is followed by no ill-results. The sole disadvantage that can be urged lies in the fact that the removal of the gall-bladder renders impossible its future anastomosis to the intestine. On the other hand, many advantages are gained; thus, the common site of the formation of gall-stones is removed, a thickened, diseased, and usually infected viscus is removed, and any chance of further trouble from disease of the cystic duct, such as was observed in our second case, is removed. It should be borne in mind, moreover, that such attacks as were observed in this case may be of a much more serious nature, and may even result in the eventual death of the patient, as has occurred in a patient in my own practice, in spite of repeated operations for their relief. Beyond these more solid advantages, the time of recovery is materially shorter than that necessitated by the operation of cholecystostomy, while the patient is spared the discomfort of the temporary discharge of bile from the fistulous opening.

The avoidance of adhesions is a more difficult matter; these are often present at the time of operation and have to be separated, while the quietude of this angle of the abdominal cavity, so favourable as regards the avoidance of widespread infection is concerned, favours the formation of adhesions. The only precautions that can be recommended are the observance of the strictest cleanliness, and the limitation as far as possible of the field of operation to the structures actually to be dealt with. These points are best dealt with by the free exposure of the parts by the use of Mayo Robson's incision, combined with rotation of the liver, gentle separation of adhesions, efficient packing off of surrounding parts, the evacuation of the gall-bladder if necessary with an aspirating syringe, and, if omentum is separated, the sewing-over of raw surfaces with normal peritoneum when this is possible.

